# A review on emerging pharmaceutical residues in Ethiopia: occurrence, ecotoxicological aspects, and regulatory concerns

**DOI:** 10.3389/fmicb.2024.1499487

**Published:** 2024-12-20

**Authors:** Addisu Afrassa Tegegne, Yesuneh Tefera Mekasha, Adugna Abera Ayu, Gemmechu Hasen, Sultan Suleman

**Affiliations:** ^1^Department of Pharmaceutical Chemistry, School of Pharmacy, College of Medicine and Health Sciences, University of Gondar, Gondar, Ethiopia; ^2^Pharmaceutical Sciences, Pharmaceutical Quality Assurance and Regulatory Affairs, University of Gondar, Gondar, Ethiopia; ^3^Department of Industrial Chemistry, College of Natural Science, Arba Minch University, Arba Minch, Ethiopia; ^4^Jimma University Laboratory of Drug Quality (JuLaDQ) and School of Pharmacy, Jimma University, Jimma, Oromia, Ethiopia

**Keywords:** pharmaceutical residues, ecosystem disruption, wastewater treatment, drug use, regulatory oversight, sustainable drug practices

## Abstract

**Background:**

Pharmaceuticals are expected to improve human and animal health, but improper management and regulation have led to adverse effects such as reproductive disorders, antibiotic resistance, and biodiversity loss in ecosystems. Their presence in the environment poses significant risks, including a reduction in biodiversity, reproductive issues, and the development of antimicrobial resistance. This review aims to examine the occurrence and sources of pharmaceuticals in the environment and their ecotoxicological and regulatory aspects, with a focus on Ethiopia.

**Methods:**

A narrative review of relevant studies conducted in Ethiopia was undertaken. The review included findings on the occurrence, sources, contributing factors, ecotoxicological impacts, and regulatory concerns related to pharmaceutical residues in the environment. Literature was sourced from Google Scholar, Scopus, PubMed, and institutional repositories.

**Result:**

The findings revealed the detection of pharmaceutical residues in wastewater treatment facilities, aquatic environments (e.g., lakes and rivers), and commercially available animal products. Aquatic samples also showed significant concentrations, with sulfamethoxazole and fluconazole detected at 0.15 μg/L and 0.012 μg/L, respectively. Antimicrobial resistance genes were identified in wastewater and treatment plant samples, which correlate with the presence of pharmaceutical residues. An ecological risk assessment based on the risk quotient (RQ) revealed ciprofloxacin as a major concern, with an RQ of 8.58, indicating high ecological risk. Sulfonamides exhibited moderate risk, with RQ values ranging from 0.1 to 1.

**Conclusion:**

The study highlights the significant presence of pharmaceutical residues in the environment and underscores the inadequacy of regulatory enforcement in addressing this public health issue. Urgent measures are required to prevent environmental contamination and mitigate public health risks, including antimicrobial resistance. Strengthened regulatory measures and proactive interventions by relevant organizations are essential to control and prevent pharmaceutical residues in the environment, offering a critical solution for the country.

## 1 Introduction

Pharmaceuticals for humans and animals play a fundamental role in retarding disease development and related complications, aggregating healthy life, and lowering mortality rates. The increasing and aging global population leads to a rise in the use and production of pharmaceuticals. Given that pharmaceuticals are intended to impact living organisms, it is well known that the lack of proper management of them can negatively affect wildlife and ecosystems (O’Flynn et al., 2021). In addition, their intensified environmental exposure puts pressure on biodiversity, leads to antimicrobial resistance, and risks our long-term health (Ortúzar et al., 2022). According to the study by Lancet (2000–2018), antibiotic consumption among humans increased significantly by 46% (Browne et al., 2021). Comparably, up to 90% of active ingredients in oral dosage formulations were excreted in the urine unchanged, which may be released directly into the environment.

In addition, in husbandry, pharmaceuticals can enter the environment easily, especially when used as a fertilizer. Such entry undergoes degradation, movement, and accumulation, which can result in long-term gloomy environmental consequences. Soil contamination and biomagnification were among the negative impacts, as pharmaceuticals can be absorbed by food crops (Gros et al., 2019). A separate route of the pharmaceutical entrance into the ecosystem is the production of active pharmaceutical ingredients and their formulations. The industrial manufacturing effluents generated while cleaning pharmaceutical ingredients and manufacturing equipment are likely the primary pathways for their release into the environment (Moermond et al., 2022).

Literature indicates that pharmaceuticals are widely present in aquatic ecosystems, where their physicochemical properties and bioactivity can result in ecotoxicological effects, even at low-to-moderate concentrations (Nguyen et al., 2024). The increasing use and subsequent release of these substances into watersheds have emerged as a global concern due to their persistence in the environment and potential adverse impacts on humans, animals, and ecosystems. Factors such as environmental conditions and chemical structure significantly influence their degradation, making many pharmaceuticals non-biodegradable (Moghaddam et al., 2023). Their persistence exacerbates issues such as antibiotic resistance, posing risks to biodiversity and human health (Cherian et al., 2023).

To address this issue, pharmaceutical facilities have adopted various problem-solving methods and technologies. Primarily, these methods focus on eliminating drug residues before pharmaceutical effluents or sludge solids are discharged into water bodies, treatment plants, and, ultimately, the surrounding environment (Samal et al., 2022). Recent advancements in environmental protection have been increasingly embraced by the biotech industry, with a particular focus on the sustainability of drug discovery. This shift represents a critical step toward developing more innovative and effective solutions to mitigate the environmental impact of pharmaceuticals (Wynendaele et al., 2021).

Globally, the occurrence of pharmaceuticals in the surrounding varies by country, which depends on their consumption and monitoring. Contamination due to the reluctant use of pharmaceuticals, poor sanitation, and inadequate wastewater treatment facilities was prevalent in Africa, which resulted in easy occurrence (Waleng and Nomngongo, 2022). Similarly, a portion of medicines can be excreted and enter the aquatic environment easily after municipal treatment. In Ethiopia, such a problem is exacerbated by the lack of waste treatment facilities in the pharmaceutical industries, as only one of them uses a septic tank with limited retention capacity (Wongiel et al., 2018). A recent review puts Ethiopia as the highest rated in Africa for contaminated samples in pharmaceutical residues, followed by Tunisia (Wilkinson et al., 2022). The substantial veterinary residue occurrence in the country can be attributed to the absence of detection facilities and standards for the maximum residual limits for drugs in food (Ame et al., 2022). This review study aimed to bring forward an overarching baseline data assessment on the occurrence, distribution, and effect of pharmaceuticals in the Ethiopian environment, including lakes and seas, by focusing on their sources and finding the contributions to their occurrence. It also provides a record of various pharmaceutical compounds with their concentrations and discusses the ecological and regulatory aspects related to these compounds to better understand the issue of long-term, low-level exposure to pharmaceutical pollution on ecosystems found in the environment.

## 2 Review methodology

The review was conducted from May to July 2024 using various online platforms to record relevant information on pharmaceutical residues, their occurrence, ecotoxicological status, and regulatory efforts across the Ethiopian region. Databases such as Google Scholar, Scopus, and PubMed were utilized to retrieve comprehensive scientific information published in English, specifically related to studies conducted in Ethiopia. Institutional repositories were also visited to avoid missing scholarly studies that might not get published in a peer-reviewed journal. Additionally, findings from other regions were included to provide comparative insights into regulatory measures and environmental protection agency activities addressing the release of pharmaceutical residues into the environment.

The literature search employed the following keywords: “*Pharmaceutical residue OR Antimicrobial*, *Environmental health*,” “*Drug-contaminated wastewater*,” “*Medicinal wastewater*,” “*Pharmaceutical wastewater*,” “*Water safety*,” “*Contaminants*,” “*Regulatory agency AND Environmental protection*,” “*Ecotoxicology OR Aquatic Environmental pollution*,” “*Wastewater treatment*,” “*Contaminant removal*,” and “*Ecotoxicity*.”

To ensure the inclusion of relevant studies, specific criteria were established. Studies focusing on the toxicity of pharmaceutical wastewater, its public health impact, and related environmental concerns were included in the review. Boolean operators were employed to refine the search results. The “AND” operator was used to narrow the search by requiring all specified terms to appear in the results, while “OR” broadened the search to include any of the specified terms, such as *antimicrobial residue*, *anti-inflammatory residue*, *antibiotic residue*, or *analgesic*. The “NOT” operator excluded results containing irrelevant terms. Data extraction and quality assessment were conducted independently by four reviewers (AT, AA, GH, and SS) to ensure methodological validity. Any disagreements were resolved through discussions with AT.

## 3 Literature search results

### 3.1 Occurrence of pharmaceuticals in the environment

Recent and previous studies have assessed the environmental occurrence of pharmaceuticals in various aquatic bodies, including lakes, rivers (Daniel et al., 2024; Hailu et al., 2024; Gezahegn et al., 2019; Abate, 2022), groundwater, wastewater, and in dairy and poultry products (Bedada et al., 2012; Agmas and Adugna, 2018; Mohammed et al., 2022; Uma and Ashenef, 2023; Abdeta et al., 2024).

A total of 13 papers were reviewed, and over 37 distinct active pharmaceutical ingredients were detected in different samples. Studies were conducted in both target and non-targeted screening of pharmaceutical occurrences. They predominantly focused on antibiotics, especially tetracyclines, ciprofloxacin, and fluoroquinolones. Other categories also include antifungals, antiparasitics, antivirals, analgesics, antipsychotics, and antiepileptics. Notably, oxytetracycline emerged as the most frequently detected active pharmaceutical ingredient, appearing in 8 of 13 included studies, followed by ciprofloxacin, sulfamethoxazole, and carbamazepine. A summary report for the occurrence of pharmaceuticals, their concentration, and the analytical methods used is presented in Table 1.

**Table 1:** Summary of the pharmaceutical residues reported in the environment with respective concentrations, analytical methods, and remark on their detected levels.

Author, year	Categories of investigated	Sample location	Analytical methods	Period	Detected pharmaceuticals/percentage/concentration	Categories of detected residues	Comment
Agmas B. (2018)	Antimicrobial residue in beef meat	Farms at Debre Tabor and Bahir Dar	Premi^®^Test/Amhara Regional Diagnostic Laboratory Center	2017	Oxytetracycline, pinstripe, and sulfonamide/76.4% of the sample The detection limit was greater than and concentrations of oxytetracycline (100 μg/kg), pinstripe (5 μg/kg), and sulfonamide (100 μg/kg)	Antimicrobials	Higher than the acceptable daily intake stated by FAO/WHO
Uma G. (2023)	Antibiotic residues in beef meat	Butcheries in Dukem and Bishoftu towns	UPLC, TQ LC/MS/ Veterinary Drug and Feed Quality Assessment Centre	2020–2021	Oxytetracycline (22.32 ± 2.38 μg/kg) Detectable levels of oxytetracycline residues by sample location were 12% at Dukem and 8% (4/50) at Bishoftu towns	Antibiotics	Levels found are all below the EU/CAC/USFDA MRLs
Hailu K. (2024)	All categories of pharmaceuticals: screening of emerging organic compounds	Awash river and its tributaries	LCMS, GCMS, LC-QTOF/Agilent laboratories, and at the NLS Environment Agency Laboratory (Starcross, United Kingdom)	2022	Chlorothiazide (0.0002 μg/L), sulfamethoxazole (0.15 μg/L), fluconazole (0.012 μg/L), thiabendazole (0.0003 μg/L), dimetridazole (0.0044 μg/L), propiconazole (0.025 μg/L), lamotrigine (0.0003 μg/L), tramadol (0.0005 μg/L), sulfanilamide (0.014 μg/L), atazanavir (0.001–0.0043 g/L), carbamazepine (0.001–0.0058 μg/L), phenobarbital (0.069 μg/L), cocaine (0.001 μg/L), ibuprofen (0.001 μg/L), diclofenac (0.0032 μg/L), acetaminophen (0.005 μg/L)	Antiprotozoal, antibiotics diuretics, anticonvulsant, antifungals, antiviral, NSAIDs, analgesics	More than 100 emerging organic contaminants were detected, including pharmaceuticals
Abdeta D. (2024)	Antimicrobial residues in red meat and kidney of beef cattle	Municipal abattoir in Nekemte town	LC-MS/MS, (UHPLC-TQ-MS/MS)/Ethiopian Agriculture Authority laboratory center	2021–2022	Tetracycline residue levels 0.12 to 463.35 μg/kg for the kidney and 0.41 to 354.55 μg/kg for the muscle 50 and 100% of samples had detectable oxytetracycline and tetracycline residues, respectively	Antimicrobial	3.33 and 10% of muscle and kidney samples have oxytetracycline residues above MRLs, respectively
Mohammed N. (2022)	Antibiotics residues in kidney and muscle of beef cattle	Municipal abattoirs in Dire Dawa and Harar	TLC, HPLC/Ethiopian drug administration and quality control authority laboratory, Addis Ababa	2018–2019	Oxytetracycline was detected in 84% of the samples Oxytetracycline in kidney and muscle ranged from 57 to 607 μg/kg and 10.14 to 435 μg/kg respectively, in samples collected at the Dire Dawa Oxytetracycline in kidneys and muscles ranged from 16 to 433 μg/kg and 6 to 435 μg/kg, respectively, in Harar town	Antibiotics	Muscle samples 22.0 and 17.8% collected from Dire Dawa city and Harar town, respectively, exceeded the MRLs for oxytetracycline
Bedada A. H. (2012)	Antibiotics residue levels in slaughtered beef cattle	slaughterhouses in Addis Ababa, Debre Zeit, and Nazareth	TLC, UV, HPLC/Ethiopian Drug Administration and Quality Control Laboratory (DACA), Addis Ababa, Ethiopia	2006–2007	71.4% had detectable levels of oxytetracycline 9.732 μg/kg to 449.65 μg/kg for kidney and 11.5 μg/kg to 429.289 μg/kg for muscle at Addis Ababa 56.037 μg/kg to 740.59 μg/kg for kidney and 3.01 μg/kg to 145.87 μg/kg for muscle at Nazareth 63.12 μg/kg to 260.56 μg/kg in the kidney and 6.68 μg/kg to 67.34 μg/kg in the muscle at Debre Zeit	Antibiotics	48.3% at Addis Ababa, 48.1% in muscles, and 0.9% in the kidney at Nazareth were above MRLs
Tola T. (2017)	Antimicrobial residues in bovine bulk milk	Dairy Farms in Bishoftu, Central Ethiopia	Microbial inhibition plat test/AAU, Veterinary Medicine, and Agriculture Laboratory	2013–2014	6.5% were found antimicrobial residues No quantification was done	Antimicrobial	From the samples analyzed for antimicrobial residue, 6.5% were positive
Abebew D. (2014)	Antibiotics residue levels in bovine bulk milk	Dairy farms in Nazareth, Ethiopia	Delvotest SP assay, HPLC/FVM, and DACA laboratory	2007–2008	Oxytetracycline residue level was 45 μg/L to 192 μg/L, and penicillin G residue levels were 0 μg/L to 28 μg/L 12% of samples had detectable levels of antibiotic residues	Antibiotics	Oxytetracycline above MRL 83.33% of antibiotic residue-positive samples, and for penicillin, G above MRLs were 16.66%
Netsere M. (2023)	Antimicrobials, residues of backyard-slaughtered broilers meat	Dairy Farms in Bishoftu, Ethiopia	HPLC-ESI-MS/MS/Animal Product and Input Quality Testing Center (API-QTC), Akaki-Kality Sub-city, Addis Ababa	2022	Sulfadiazine (9.25 to 13.29 μg/kg), oxytetracycline (9.92 to 135.36 μg/kg), and enrofloxacin (17.38 to 943.5 μg/kg) 39.2% of samples contained detectable levels of residues; 3.3, 18.3, and 25% contained detectable levels of residues of sulfadiazine, oxytetracycline, and enrofloxacin, respectively	Antimicrobials	21.67 and 0.8% of the samples, respectively, had oxytetracycline and enrofloxacin above the EU MRL
Abate A. T. (2020)	Antibiotic residues of tannery wastewater	Wastewater from Batu Tannery at Little Akaki River	HPLC-MS/Institute of Biotechnology and Bioinstrumentation laboratories, Addis Ababa University	2015–2017	64.3% of the targeted antibiotic residues were present in the tannery wastewater samples Oxytetracycline, tetracycline, penicillin G, trimethoprim, sulfamethoxazole, sulfadiazine, sulfadoxine, erythromycin, ciprofloxacin	Antimicrobials	All of the nine antibiotic residues were uniformly detected in all tannery wastewater samples
Mamo S. (2018)	Antibiotic residues in chicken meat	Commercial poultry farm in Bishoftu town	HPLC/Kality Animal Products Veterinary Drugs and Feed Quality Assessment Center	2016–2017	Not detectable	Not detectable	Not detectable
Ijo T. G. (2018)	Antibiotics in wastewater	Pharmaceutical effluent in Addis Ababa	HPLC-DAD/	N/A	Concentrations of ciprofloxacin were 0.82 ± 0.5 μg/mL from Addis Ababa Sewage Treatment	Antibiotics	The presence of ciprofloxacin in ASTP indicates that the potential of APIs in wastewater
Daniel W. B. (2024)	Analgesics, anticonvulsants, antipsychotics, antidepressant, antianxiety, antibiotics, antifungal, anti-malarial, antiretroviral, cardiovascular, and antidiabetics	Samples of water and sediment from Lake Hawassa, Ethiopia	UHPLC-MS/MS/laboratory at Hawassa University, Ethiopia		Surface water sample: ciprofloxacin (630 ng/L), sulfonamide (2 ng/L), tramadol (89 ng/L), paracetamol (48 ng/L), ketoprofen (11 ng/L), diclofenac (16 ng/L), artemether (18 ng/L), dihydroartemisinin (8 ng/L), artesunate (7 ng/L), mefloquine (5 ng/L), phenytoin (3 ng/L), lorazepam (1 ng/L), carbamazepine (1 ng/L), flupentixol (19 ng/L), fluconazole (5 ng/L), oxazepam (5 ng/L)	Antimalarial, analgesics, antianxiety, anticonvulsants, antifungal, antipsychotic, antibiotics	Antibiotics contributed about 73% of APIs, followed by analgesics (19%) Ciprofloxacin exhibited (RQ ≥1; 8.58) Diclofenac, sulfonamide, lorazepam, mefloquine, and lumefantrine, showed (0.1 ≤ RQ < 1)
					Pooled water sample: ciprofloxacin (362 ng/L), sulfonamide (2 ng/L), tramadol (73 ng/L), paracetamol (27 ng/L), ketoprofen (20 ng/L), diclofenac (11 ng/L), artemether (14 ng/L), dihydroartemisinin (6 ng/L), artesunate (5 ng/L), mefloquine (7 ng/L), phenytoin (1.77 ng/L), lorazepam (2 ng/L), carbamazepine (1 ng/L), flupentixol (8 ng/L), fluconazole (3 ng/L), oxazepam (10 ng/L)		

#### 3.1.1 Occurrence of antimicrobial residue in the aquatic environment

Antimicrobial occurrence in aquatic environments is mainly due to the waste generated from municipalities, hospitals, pharmaceutical industries, agriculture, and aquaculture. In addition, antibiotics are well known to be excreted in urine and feces, either as active compounds or their metabolites (Kümmerer, 2009). In Ethiopia, wastewater treatment facilities and waste management systems lack the capacity to effectively remove antibiotic residues effectively (Adugna, 2023). Along this, antibiotic residues have been detected in various aquatic bodies, including rivers, lakes, groundwater, wastewater, and drinking water.

Antibiotics and antifungals (sulfamethoxazole, fluconazole, thiabendazole, dimetridazole, propiconazole, and sulfanilamide) were among the detected antimicrobials in the Awash River basin in Ethiopia (Hailu et al., 2024) (Table 1). In the aquatic samples, sulfamethoxazole is the most abundant antimicrobial detected, with a concentration of 0.15 μg/L, followed by fluconazole at 0.012 μg/L, thiabendazole at 0.0003 μg/L, dimetridazole at 0.0044 μg/L, propiconazole at 0.025 μg/L, and sulfanilamide at 0.014 ng/L (Hailu et al., 2024). It has been shown that the main reason for the increased occurrence level is the disposal of waste from the municipal and industrial sources in Addis Ababa, which is greater than the downstream areas. In Ethiopia, a remarkable portion of tannery wastewater is released directly into the water bodies with minimal or no treatment. On account of this, antibiotic residues of tetracycline, oxytetracycline, erythromycin, penicillin G, trimethoprim, sulfadiazine, sulfadoxine, sulfamethoxazole, and ciprofloxacin have been found in all samples of tannery wastewater from Batu Tannery (Abate, 2022).

A distinct study showed the residence of ciprofloxacin at a concentration of 0.82 ± 0.5 μg/mL from Addis Ababa Sewage Treatment. This might be due to the insufficient treatment facilities for wastewater disposal in Ethiopia, and the study noted that other pharmaceutical pollutants might be found in other wastewater systems (Gezahegn et al., 2019). Additionally, water and sediment samples from Lake Hawassa, Ethiopia, contained ciprofloxacin (630 ng/L) and sulfonamide (2 ng/L), along with other antibiotics and antifungal agents. Ciprofloxacin’s presence at high levels is linked to its heavy use in Ethiopia, which is estimated at 700 tons each year (Daniel et al., 2024).

#### 3.1.2 Occurrence of antibiotic residues from livestock farming: milk and meat

The studies conducted to assess the presence of antimicrobials in milk and meat also highlighted their use in food-processing animals. Such veterinary pharmaceuticals were commonly used to prevent infections in livestock management. The most detected antibiotics were oxytetracycline, tetracycline, pinstripe, sulfonamide, penicillin G, sulfadiazine, and enrofloxacin from the samples collected at various regions of Ethiopia (Table 1). Oxytetracycline residue concentration was the highest at 607 μg/kg, next to enrofloxacin at 943.5 μg/kg (Mohammed et al., 2022; Netsere, 2023). Interview findings from farmers indicated that the most used pharmaceuticals in the study area were oxytetracycline, sulfonamides, pen-strep, penicillin, procaine penicillin, ivermectin, albendazole, tinidazole, multi-vitamins, and acaricides (Table 2).

**Table 2:** Commonly used pharmaceuticals and respondent’s knowledge, practice on antimicrobial use, and drug residues.

Author, year	Commonly used pharmaceuticals in the study area	Respondent’s knowledge, practice on antimicrobial use, and drug residues
Agmas B. (2018)	Oxytetracycline; 97.67% of farms, β-lactams (pinstripe); 9.30%, and sulfonamides; 48.84% (sulfadimidine and diminazene aceturate)	All respondents of beef cattle farmers did not respect the drug withdrawal period, and drugs were given by nonprofessionals (48.84%) Lack of awareness (86.05%) and there might be negligence in withdrawal time
Abdeta D. (2024)	Oxytetracycline was indiscriminately used in the study areas	Respondents knew about drug residues, 52.50% of them 74% of the respondents know about the drug withdrawal period and its health effects Only 40% of respondents know methods to control and prevent drug residues 58.33% of participants disagreed that antibiotics may end up in animal tissue Only 60.83% thought that heating meat could lower the possibility of antibiotic residue
Abebew D. (2014)	Oxytetracycline (46.74%), pinstripe (36.96%), and multijet antibiotics (18.48%)	35.87% of dairy farm owners had received training in dairy management In 21.74% of the farms, antibiotics were administered by a veterinarian 63.04% of farms reported withholding milk from all quarters of treated cows No farms that used the antibiotic residue test kit Approximately 60% knew that antibiotic residues were of public health significance
Abate A. T. (2020)	Oxytetracycline, sulfonamides, penstrep, penicillin, procaine penicillin, ivermectin, albendazole, and tinidazoles	Problems with inappropriate withdrawal of food animals after administration Lack of awareness of the environmental side effects of antimicrobials by the farmers, but (76%) showed that there was little concern about the environmental consequences of animal wastes with which residual antibiotics
Mamo S. (2018)	Tetracycline and oxytetracycline, amoxicillin, and amprolium	Workers are not even aware that drug residue in meat can affect human health Managers and professionals know about residues and their risk to human health, but they have not put in place protocols for feeders to prevent residue Respondents have no withdrawal time for oxytetracycline
Beyene T. (2015)	Oxytetracycline (100%), amoxicillin (71.4%), ciprofloxacin (28.6%), amprolium (100%) and sulfa drugs (26.3%), piperazine (31.0%), albendazole (9.1%) and tetramisole (7.1%)	70.0% of farms sell their product before the withdrawal period 59% have awareness and information about chemical contamination of animal feed 57.1% of them prefer professionals who have a doctor of veterinary medicine (DVM) and above to treat sick animals
	Ivermectin, oxyclozanide, and tetramisole, penicillin-streptomycin combination (pen strep) injection, oxytetracycline injection, and ampicillin cloxacillin combination (intramammary infusion)	About 60.0% of farms sell their animal products (milk) while their animals are under treatment 46.7% preferred veterinary doctors and above professionals Only 66.7% of dairy farms are aware of drug residue
Tadele M. (2022)	Albendazole and triclabendazole, imidazothiazole (tetraclozan and tetramisole/levamisole) and macrocyclic lactone (ivermectin) classes were common in all districts. The most frequently used antibiotics are tetracycline (oxytetracycline), β-lactams (penicillin-streptomycin), and sulphonamides (sulfadimidine)	53.3% of the respondents adhere to the recommended drug withdrawal period Only 36.7% of the farmers get drugs using prescriptions 42.2% have an awareness of the risk of AMR in milk

Findings indicated that roughly 3.33% of muscle samples and 10% of kidney samples from Nekemte town accommodated oxytetracycline residues above the maximum residual limits. The muscle samples collected from Dire Dawa city showed 22.0%, while those from Harar had 17.8%. In Addis Ababa, 48.3% of muscle samples exceeded the limits; in Nazareth, the figure was 48.1%. Additionally, 83.33% of milk samples from Nazareth and 21.67% of broiler meat in Bishoftu had residues above acceptable levels, which could pose health risks to public health (Table 1).

#### 3.1.3 Occurrence of analgesics, anti-inflammatory drugs, and other categories

The studies reveal the presence of analgesics and anti-inflammatory drugs in samples from rivers, wastewater, and industrial effluents. It has been widely acknowledged that non-steroidal anti-inflammatory drugs, even at concentrations below μg/L, are considered hazardous emerging pollutants that can ultimately impact aquatic environments (Tyumina et al., 2020). Analgesic categories of pharmaceuticals detected in the current literature include tramadol, ibuprofen, diclofenac, acetaminophen, and ketoprofen (Daniel et al., 2024; Hailu et al., 2024). Furthermore, atazanavir was identified at concentrations between 0.001 and 0.0043 g/L.

Samples of water and sediment from Lake Hawassa, Ethiopia, showed that various pharmaceutical residues, including anticonvulsants, antipsychotics, antidepressants, antianxiety medications, antimalarials, antiretrovirals, cardiovascular drugs, and antidiabetics, were detected. From the surface water sample, detected residues include artemether (18 ng/L), dihydroartemisinin (8 ng/L), artesunate (7 ng/L), mefloquine (5 ng/L), phenytoin (3 ng/L), lorazepam (1 ng/L), carbamazepine (1 ng/L), flupentixol (19 ng/L), and oxazepam (5 ng/L) (Daniel et al., 2024).

### 3.2 Pharmaceutical sources and pathway of entry to the environment

Pharmaceutical residue occurrence in the environment comes at various stages of their product lifecycle, from production and consumption to disposal (O’Flynn et al., 2021). Their sources include wastewater from industries, hospitals, and households, improper disposal of medications, and livestock treatment emissions. Undoubtedly, patients’ utilization of prescription and over-the-counter medications has been taken as a notable source, as they are excreted and easily enter waterways after municipal treatment (Kümmerer, 2009). It has been known that human-consumed medicines reach the environment through direct discharges from municipal wastewater treatment plants (Aydin et al., 2019). In addition, pharmaceuticals also find their way into the environment through agricultural and veterinary practices. In Ethiopia, there is minimal oversight from government authorities, and there is a lack of information regarding rational drug use in veterinary practices. Moreover, animals slaughtered in the country are not tested for drug residues in any slaughterhouses, and the waste generated is not regulated and treated for the benefit of the environment (lack of a greenery approach). Consequently, pharmaceuticals can directly enter the ecosystem during livestock disease management practices by farmers (Wongiel et al., 2018).

#### 3.2.1 Residential households

Household use of pharmaceuticals can be a cause for their entrance into the environment as a result of home consumption. Evidence shows that in low-income countries, feces and urine with excreted pharmaceuticals often end up in pit latrines, septic tanks, or the environment due to open defecation (Gwenzi et al., 2023). Previous studies showed that household consumption of prescribed and non-prescribed pharmaceuticals is considered the primary source of the environment (Kümmerer, 2009). It is also reported in Ethiopia; currently, beef fattening farms use drugs irrationally in frequent and overdose ways that result in a high burden of veterinary residues. In addition to excreted drugs, a household’s improper disposal of pharmaceuticals can raise drug levels. This happens when people throw away expired or unused medications down sinks, toilets, or with regular trash that goes to landfills. How much these drugs enter the environment depends on how patients dispose of them and how well prescriptions are managed to reduce leftovers (Rogowska and Zimmermann, 2022). In developing countries, including Ethiopia, liquid pharmaceuticals are often flushed down toilets and sewer systems. Among others, in Ethiopia, the community’s knowledge of proper disposal of unused medications was found to be 42.6% (Tegegne et al., 2024). Unused medicines disposed of in garbage bins (63%) are the most common practice in Ethiopia (Kahsay et al., 2020), and the lack of a drug take-back system may exacerbate pharmaceutical pollution in the environment.

#### 3.2.2 Hospitals/health facilities

Pharmaceuticals were also found in health facilities, as they are known for dispensing and using a wide range of therapeutics, which makes them a source of drugs that enter municipal waterways. It was believed that health facilities have lower pharmaceutical levels than household and industrial waste, and the predominance of rural hospitals in Ethiopia has made the problem more concerning. This is because rural-based hospitals often have smaller, less advanced wastewater treatment plants (WWTPs), which are likely less efficient in retaining pharmaceutical residues (Manderso, 2018). It has been reported in Ethiopia that 68% of respondents from health facilities agreed that current pharmaceutical waste management practices are unsatisfactory and have an impact on the environment. Specifically, 16.3% of pharmaceutical waste was disposed of in open landfills, 63.7% was burned openly, and 20% was treated through incineration (Mohammed et al., 2021). In Ethiopia, there are water bodies in the vicinity of healthcare facilities that can cause easy entrance of the facilities’ effluent. To illustrate, a freshwater lake in Ethiopia, Lake Hawassa, is situated near the Hawassa University Comprehensive Specialized Hospital on its eastern shore. Consequently, this lake receives both wastewater and healthcare waste, which may include active pharmaceutical ingredients (Daniel et al., 2024).

#### 3.2.3 Pharmaceutical industries/manufacturing facilities

Pharmaceutical production facilities raise significant concerns due to their extremely high discharge concentrations, which contribute to point source pollution (Larsson et al., 2007). Countries such as China, Korea, and India have found that treated wastewater from pharmaceutical industries contains high levels of hygromycin, lincomycin, and fluoroquinolone antibiotics, measured in milligrams per liter (Milaković et al., 2019). Significant water is required during pharmaceutical production, which results in the generation of a substantial amount of wastewater. Additionally, untreated wastewater released into the environment poses a serious risk to ecosystems and human health. This issue is prominent in developing countries with no effective treatment facilities for industrial effluents (Nidheesh et al., 2022). A 2018 survey indicated that the pharmaceutical manufacturing industry in Ethiopia does not utilize high-temperature incineration or waste immobilization methods. As a result, the water used for cleaning during production contains dissolved or suspended medications that are released into the effluent without any prior treatment. Furthermore, nearby vegetable farms that source their water from industrial effluents may expose residents to small quantities of pharmaceuticals by consuming raw vegetables grown in that area (Wongiel et al., 2018).

#### 3.2.4 Agriculture and aquaculture

Veterinary pharmaceuticals enter aquatic ecosystems directly from agriculture and aquaculture activities. Farmers and veterinarians in low-income countries experiencing food insecurity often struggle to obtain effective antibiotics for treating their livestock. However, disruptions in access to these pharmaceuticals lead to environmental contamination, as many individuals lack training and do not adhere to the proper withholding periods after treatment (Grace, 2015). The problem of antibiotic residues in meat is significant and not adequately managed in low- and middle-income countries, including Ethiopia. In Ethiopia, food animals intended for domestic and export markets are not tested for residue presence in any slaughterhouses.

Additionally, it has been reported that oxytetracycline is used indiscriminately in various regions of the country (Abdeta et al., 2024). It is known that aquaculture has been experiencing prominent and rapid growth among food-producing sectors and has become a strong and essential global industry. However, it has also been shown to potentially cause serious environmental harm (Lai et al., 2018). In Ethiopia, despite abundant freshwater and fish resources, favorable climatic conditions, dedicated labor, and high local demand for fish, aquaculture development lags behind other agricultural activities (Chimdo, 2022). The country currently exercises freshwater aquaculture, both in fishponds and fish pens or fish cages.

### 3.3 Factors contributing to pharmaceutical entry into the environment

#### 3.3.1 Location and wastewater treatment infrastructure

Inadequate wastewater treatment facilities that allow untreated industrial wastewater to be discharged and runoff from agricultural activities have been linked to the environmental occurrence of pharmaceutical residues. This problem is noteworthy when the aquatic bodies are often located near these industrial wastewater discharges, including those from pharmaceutical manufacturing. The aforementioned issue is particularly acute in Addis Ababa, Ethiopia. Over 90% of industries in Addis Ababa release their waste directly into nearby rivers without proper wastewater treatment (Yohannes and Elias, 2017). This significantly contributes to pharmaceutical contamination in the region and creates serious environmental and public health concerns. Moreover, only one pharmaceutical industry in Ethiopia has a waste treatment facility, despite the mandatory requirement for these industries to prevent the discharge of toxic materials into the sewer system (Wongiel et al., 2018). The study conducted in Hawassa revealed that the direct inflow of untreated wastewater significantly impacted the most polluted areas. Primarily, this wastewater source carries active pharmaceutical ingredients from Hawassa University Comprehensive Specialized Hospital, which currently operates a waste treatment system that is not functioning properly. This, in turn, exacerbates the pharmaceutical pollution issue in the surrounding environment (Daniel et al., 2024).

#### 3.3.2 Disease status, variability in drug use, and withdrawal periods

Pharmaceutical residue presence in animal products may result from the subtherapeutic, prophylactic, or therapeutic use of antibiotics that stems from a lack of awareness and inadequate educational outreach (Mohammed et al., 2022). This, in turn, comes from the misuse and overuse of these drugs, as well as a failure to adhere to withdrawal periods. An Ethiopian study showed that 25.8% of respondents were unaware of the withdrawal period and its potential public health impacts. Additionally, 60% of respondents lack knowledge about different methods to control and prevent drug residues in husbandry (Abdeta et al., 2024). The pharmacokinetics of a drug can be influenced by animals’ health conditions, which, in turn, leads to the accumulation of pharmaceuticals in the affected tissues or organs. During clinical mastitis, for instance, ketoprofen levels in milk rise due to the influx of serum components into the udder (Beyene, 2016).

Moreover, residues can occur due to the indiscriminate use of antibiotics for treating infectious diseases, such as clinical mastitis and viral infections in veterinary practices. Off-label drug use involves using medications for unapproved conditions or administering them at doses higher than recommended, both of which have also been associated with residue occurrence (Ture et al., 2019). Inadequate identification of treated cows is one reason dairy farmers fail to recognize them and withhold milk from the market until the appropriate withdrawal period. Treated animals should be identified, and antibiotic usage must be documented to prevent residues. However, only about 20% of dairy farms in Nazareth, Ethiopia, maintain records of antibiotic treatments. Consequently, according to the study, insufficient record-keeping and a lack of knowledge about withdrawal periods among producers were identified as significant contributors to drug residues in milk (Abebew et al., 2014).

#### 3.3.3 Households’ improper disposal practice of pharmaceuticals

Low-income countries often struggle with insufficient solid waste and wastewater management systems, limited environmental monitoring, and a lack of knowledge about global best practices for the use and disposal of hazardous wastes, such as organized take-back programs for unused and expired medications (Gwenzi et al., 2023). Among the pharmaceutical residues detected at high concentrations, 38% were in a sample collected from a site affected by untreated domestic storm-water runoff (Daniel et al., 2024). This issue may be due to the uncontrolled, open dumpsites practiced in developing nations, including Ethiopia. In addition, a study in Hawassa, Ethiopia, found that expired or unused medication disposal in household garbage bins is a common practice practiced by over two-thirds of the respondents from households (Woldamicael Bekele et al., 2023). This improper disposal of pharmaceuticals in open, non-engineered landfills exposes scavengers to risks and allows pharmaceuticals to leach into water sources.

### 3.4 Ecotoxicological aspects of detected pharmaceuticals

Pharmaceuticals are active substances that can persist in the environment and are considered to be a threat to ecological balance. The ongoing use of these drugs, even at a small, sub-therapeutic level, can pose risks to public health. In turn, many non-target organisms with similar metabolic processes, receptors, or molecules to humans and animals may unintentionally be exposed to these active substances released into the environment (Isidori et al., 2005).

Pharmaceutical pollution poses a significant threat to both aquatic and terrestrial ecosystems. When improperly disposed of, pharmaceuticals can enter the environment through various pathways, such as wastewater treatment plants, landfills, and agricultural runoff. Once in the environment, these compounds can directly harm organisms, disrupt their endocrine systems, and contribute to the development of antibiotic resistance (Samreen et al., 2021). Additionally, pharmaceuticals can accumulate in the tissues of organisms, leading to biomagnification and posing a greater risk to higher-level predators. The long-term effects of pharmaceutical pollution can include ecosystem disruption, biodiversity loss, and potential health risks to humans. To mitigate these negative impacts, it is crucial to implement effective waste management practices, promote responsible pharmaceutical use, and develop innovative technologies for treating contaminated water and soil (Yohannes and Elias, 2017).

#### 3.4.1 Toxicity to aquatic organisms

According to a study in Ethiopia, certain pharmaceutical residues were found in a concentration above the maximum residual limits, potentially leading to their accumulation in the aquatic system. Spanning from antibiotics to hormone medicines, it can have acute and chronic toxicity on aquatic life, leading to biodiversity alteration and misfunctioning of ecosystems (Oliveira et al., 2015). Various antibiotics have been detected in surface waters and groundwater in Ethiopia in recent years, including ciprofloxacin, oxytetracycline, tetracycline, erythromycin, penicillin G, trimethoprim, sulfamethoxazole, sulfadoxine, and sulfadiazine.

The ecological risk assessment was based on the risk quotient (RQ) value, calculated from the highest concentrations of detected pharmaceuticals in water and sediment samples from Lake Hawassa. It was ciprofloxacin, among the antibiotics, with an RQ of 8.58, indicating a prominent ecological risk to aquatic life, similar to algae. In addition, sulfonamide also showed a moderate ecological risk, with an RQ of 0.1 to 1 (Daniel et al., 2024). A prior study found that exposure to ciprofloxacin resulted in marked decreases in both the growth rate and cell density of microalgae compared to control groups (Diniz et al., 2021). This review identified oxytetracycline and sulfamethoxazole as detected antibiotics, shown in prior research, to impact the growth of *M. aeruginosa* and *C. microsphaera* (Zhou et al., 2021). The antibiotic oxytetracycline functions by disrupting aminoacyl-tRNA binding in ribosomal protein synthesis in bacteria (Chukwudi, 2016). It was also known that it is not effectively removed in wastewater treatment plants, but it is recognized for its fortuitous effect on non-target aquatic organisms (Kovalakova et al., 2020).

Sulfadiazine was another frequently detected antibiotic in the studies reviewed, and it was recognized for its low removal rate in conventional wastewater treatment processes. Recent research indicates that sulfadiazine alone inhibited the growth of microalgae, with the inhibitory effect increasing at higher concentrations (Li et al., 2022). The antiviral drug atazanavir was also detected. Due to their harmful effects on algae, Daphnia, and fish, antiviral drugs have been identified as some of the most dangerous and toxic pharmaceuticals (Zhou et al., 2015). The EC50 values for *V. fischeri* classify both ciprofloxacin and oxytetracycline as acute aquatic toxicity category 3. The analgesic drug ibuprofen was another detected residue in water bodies, identified primarily for its toxic potential in aquatic living organisms. Ibuprofen’s high lipophilicity and low biodegradability nurture its bioaccumulation in the environment, and its biological effects indicate that it is detrimental to various aquatic species (Oliveira et al., 2015).

A study conducted in Ethiopia revealed that mefloquine, lumefantrine, and lorazepam had an RQ greater than 0.1 in sediment, indicating a moderate risk to the aquatic ecosystem of Lake Hawassa. Lumefantrine is known to have variable effects on the growth of the aquatic plants studied. Low concentrations can induce oxidative stress and affect the growth of chlorophytes and aquatic macrophytes. Previous research demonstrated a decrease in the growth of *R. subcapitata* and *L. minor* following exposure to lumefantrine (Chia et al., 2021). Artemether was another antimalarial found to be detectable, which is the most commonly prescribed drug for the treatment of acute uncomplicated malaria. However, it is only slightly soluble in water and may not be easily degradable in the aquatic environment.

#### 3.4.2 Toxicity to terrestrial organisms

Antimicrobial pharmaceutical utilization in animals, even at low doses, is contemporaneous with the development of bacterial resistance and potential cross-resistance with human antibiotics. Thereupon, they can harm terrestrial ecosystems through nitrifying bacteria and inhibit the growth of crops through bioaccumulation. Additionally, antibiotics present in sewage treatment influents can disrupt bacterial treatment processes and lead to toxic effects in terrestrial ecosystems at various levels of the food chain. Furthermore, animals excrete it in their manure, which adversely impacts the survival and reproduction of organisms that break down manure, including dung beetles and entomopathogenic nematodes (Daghrir and Drogui, 2013).

Studies have shown that calculations of the risk quotient (RQ) for oxytetracycline and ciprofloxacin indicate medium to high adverse effects on bacteria and other soil organisms (Parente et al., 2018). In addition, erythromycin was detected in a study conducted in Ethiopia. A prior study indicated that erythromycin induced acute toxic effects on the photosynthesis of *Selenastrum capricornutum* at a concentration of 0.06 mg/L. It was noted that this antibiotic significantly disrupted key physiological processes, including primary photochemistry, electron transport, photophosphorylation, and carbon assimilation (Liu et al., 2011). A commonly prescribed anticonvulsant, carbamazepine, was also detected in the environment in this review. It is the most researched emerging pharmaceutical in terrestrial systems, primarily because of its low removal efficiency in wastewater treatment plants. It sometimes exhibits negative removal efficiency without seasonal concentration variations (Koba et al., 2016). The non-steroidal anti-inflammatory drug diclofenac has also been found in Ethiopia, as it is recognized for causing significant ecological harm in the sudden decline of vulture populations. Such episodes of decline in vulture populations led to both biological and social consequences for the affected region, as the absence of these scavenging birds allowed harmful bacteria and infections to spread (Ogada et al., 2012).

#### 3.4.3 Toxicity in humans

On average, each person in Addis Ababa consumes approximately 3 kg of chicken meat per year (Dessie et al., 2003; Mesele, 2023). The presence of pharmaceutical residues in animal products (like eggs, meat, and milk) intended for human use is a significant concern for public health. Therefore, foods of animal origin must be safe and nutritious for human consumption. The study revealed that oxytetracycline was found in the kidneys and muscles of slaughtered cattle sold in Nekemte town, with up to 10% of the samples exceeding the recommended maximum residue limits (Abdeta et al., 2024). In samples from Nazareth dairy farms in Ethiopia, 83.33% had oxytetracycline above the maximum residual limits, and 16.66% also exceeded penicillin G (Abebew et al., 2014). It was associated with health risks in humans from consuming chicken tissues containing oxytetracycline residues (Darwish et al., 2013). The possible menace includes the transmission of antibiotic-resistant bacteria that are known foodborne pathogens (such as *Salmonella* spp., *Escherichia coli*, and *Campylobacter* spp.), immunological effects, disruption of intestinal microflora, and inherent carcinogenicity (Lawal et al., 2015).

Slaughtered broiler meat at dairy farms in Bishoftu, Ethiopia, found that 0.8% of samples contained enrofloxacin levels exceeding the EU maximum residue limit (Netsere, 2023). It is known that enrofloxacin disrupts bacterial DNA gyrase and has been previously shown to cause teratogenic effects in rabbit and rat embryos (Guzmán et al., 2003). Comparably, 58.33% of participants in Nekemte town disagreed that antibiotics used in animals end up in their tissues. On the other hand, only 52.50% were aware of drug residues in slaughtered animals (Abdeta et al., 2024). This lack of awareness in Ethiopia could increase the population’s risk of antibiotic residue contamination.

#### 3.4.4 Development of antimicrobial resistance

Antimicrobial resistance (AMR) is a pressing global health crisis. It occurs when microorganisms, such as bacteria, viruses, fungi, and parasites, develop resistance to the drugs designed to kill or control them. This resistance can make infections more difficult to treat, leading to increased morbidity, mortality, and healthcare costs. Several factors contribute to AMR, including the overuse and misuse of antibiotics, poor infection prevention and control practices, agricultural use of antibiotics, and the spread of resistant bacteria through travel and trade (Beyene et al., 2023). Although antibiotic residues in food can lead to allergic reactions, teratogenicity, mutagenicity, and carcinogenicity, the most consequential concern is their augmentation of antibiotic resistance. Multi-drug-resistant strains of microbes have been considerably reported in soil and aquatic environments, predominantly due to the poultry and veterinary sectors’ pharmaceutical uses, which increases the prospect of bacteria surviving under antibiotic pressure (Samreen et al., 2021). To address AMR, it is essential to implement strategies that reduce the overuse and misuse of antibiotics, improve infection prevention and control measures, develop new antibiotics, and promote antimicrobial stewardship.

A study conducted at Bishoftu farms in Ethiopia assessed pharmaceutical residues and examined the antimicrobial resistance profiles of 20 *Salmonella* isolates and 27 *E. coli* O157:H7 isolates (Netsere, 2023). The results showed that at least one class of antibiotic was present in 19 (95%) of the *Salmonella* isolates and 26 (96.3%) of the *E. coli* O157:H7 isolates. Among these, 13 (65%) of the *Salmonella* isolates and 11 (40.7%) of the *E. coli* O157:H7 isolates were classified as multidrug-resistant. Additionally, 3 (15%) of the *Salmonella* isolates were prominently drug-resistant, and 1 (5%) was pan-drug-resistant. Antibiotic residues and resistance genes in wastewater from Batu Tannery, which is released into the Little Akaki River in Ethiopia, were also investigated (Abate, 2022). The specific genes tested for using PCR include tet (A), te t(O), tet (M), tet (Q), erm (B), Sul (I), Sul (II), Otr (A), and qnr (A). Accordingly, the results showed that all targeted genes were present in some wastewater samples except for tet (Q). Therefore, the current wastewater treatment method does not effectively remove antibiotics and resistance genes.

### 3.5 Pharmaceuticals in the environment: regulatory concerns in Ethiopia

To protect the environment and public health from potentially harmful exposures, it is important to regularly monitor pharmaceutical residues in the environment. Regulatory actions should be initiated when elevated concentrations are detected. Several countries have already provided evidence of the feasibility of monitoring and addressing pharmaceutical residues at various levels. In Ethiopia, pharmaceutical waste production from public and private healthcare facilities, pharmaceutical companies, households, and home care settings has risen sharply. This waste management issue is a significant concern for regulatory authorities. Since 1990, the Ministry of Health has overseen the regulation of healthcare waste in the country (Hygiene and Environmental Health Directorate of the Federal Ministry of Health, 1990). However, much of this waste is frequently mishandled, often ending up in municipal landfills, incinerated at low temperatures, or burned in open areas. This could include expired, unused, and contaminated medications and vaccines, which are classified as pharmaceutical wastes. Waste management in the country is disorganized, unprofessional, and not aligned with established rules and regulations (Abebe et al., 2023).

The Medicines Waste Management and Disposal Directive, enacted by the Ethiopian Food and Drug Authority (EFDA) in August 2011, aims to safeguard public health and the environment from risks associated with improper management and disposal of medicines. However, findings indicate that government oversight of waste generation is limited, with only 43% of respondents from the pharmaceutical industry and import sector reporting that their waste is regulated (Wongiel et al., 2018). Additionally, there is confusion regarding responsibility for waste management, as some stakeholders believe that regulation falls under both the Ethiopian Environmental Protection Agency (EPA) and the EFDA.

Ethiopia’s Federal Environmental Protection Authority (EPA) regulates activities that harm the environment. Its 1997 policy emphasizes community education, public-private partnerships, and modern waste minimization and recycling practices. Moreover, the Hygiene and Environmental Health Directorate of the Federal Ministry of Health created a healthcare waste management manual in 2008, updated in 2021, which outlines methods for managing pharmaceutical waste in healthcare facilities (Hygiene and Environmental Health Directorate of the Federal Ministry of Health, 2021). Recommended practices include returning waste to suppliers, encapsulation, dilution, sewer discharge for small amounts, incineration, and sanitary landfills for non-hazardous waste. It also stresses minimizing antibiotics and pharmaceutical residues in wastewater. Despite federal guidelines, there are no specific rules for managing hazardous and healthcare waste at the micro level. This indicates that sub-city health offices responsible for waste management are not paying enough attention to healthcare waste and lack proper supervision. Additionally, there are challenges, such as the healthcare waste management policy not being reviewed and weak enforcement of laws in healthcare facilities. It also stresses minimizing antibiotics and pharmaceutical residues in wastewater in the manual. However, the agencies overseeing wastewater management, including various ministries and the Environmental Protection Authority (EPA), lack clarity about their roles (Abebe et al., 2023).

Despite Ethiopia having fewer industries, factories still discharge effluents into lakes and rivers, posing significant challenges in balancing industrial development with pollution control For instance, the establishment of the Kilinto Pharmaceutical Industrial Park, aimed at reducing reliance on imports, has underscored the need for comprehensive environmental assessments and effluent treatment systems (Ababa, 2017). The industrial park’s toxic waste of discarded medications and medical waste from the pharmaceutical manufacturing industries particularly impacts the heavily polluted Akaki River in Addis Ababa (Abate, 2022). Fortunately, this indicates that responsible institutions must enforce a strong regulatory framework to ensure compliance with environmental policies (Yohannes and Elias, 2017). This is crucial for fostering socially responsible and eco-friendly sustainable manufacturing industries, which are expected to develop through industrial parks.

In many African countries, including Ethiopia, there is a lack of clear regulations to control antibiotic contamination and residues in husbandry (Darwish et al., 2013). Ethiopia’s Federal Constitutional Decree No. 728-2011 mandates that only veterinarians are authorized to prescribe veterinary medicines; however, diseases are often inadequately treated, with only 36.7% of farmers obtaining prescriptions (Tadele et al., 2022). This highlights challenges in regulatory enforcement, resulting in the irrational use of drugs without proper diagnoses, which ultimately affects the environment. Furthermore, Ethiopia’s current food legislation lacks standards addressing antibiotic residues, despite the widespread use of antibiotics among livestock producers. The country has faced difficulties in establishing effective standards and best practices for safe food production, especially regarding antimicrobial withdrawal times and maximum residue limits. The National Codex Contact Point of Ethiopia (NCCP-Ethiopia) is tasked with adopting standards, such as Ethiopian Standards, and implementing technical regulations in specific areas. Moreover, an integrated national plan, the Integrated National Antimicrobial Resistance and Residue Surveillance Plan in Animal Health, Plant, Food Safety, and Environment Sectors of Ethiopia (2019–2023), aims to address antibiotic waste across various sectors (Food and Agricultural Organization, 2019). However, the lack of sustainable investment, unaccredited quality control tests, and inadequate monitoring of drug residues in animal-derived food are significant limitations to effective regulatory implementation (Beyene et al., 2023). Research has shown that areas with less stringent regulations, particularly in African countries where antibiotics can be purchased over the counter, experience higher levels of pharmaceutical residue in rivers (Wilkinson et al., 2022). This highlights how weak regulation of antibiotic dispensing contributes to improper human and veterinary use, allowing these substances to enter aquatic systems more easily. The Medicines Waste Management and Disposal Directive (MWMDD) seeks to minimize the environmental impact of medicines waste. To achieve this, countries such as Ethiopia and the majority of African countries must implement strategies to prevent and minimize waste generation, establish efficient collection systems, utilize innovative disposal technologies, and monitor and report compliance. Optimizing prescribing practices, educating patients, and improving drug formulations can further reduce medicine waste. Additionally, implementing clear labeling, secure transportation, and proper disposal methods can help prevent the illegal disposal of medicines waste. Regular monitoring and reporting are essential to ensure compliance with the directive and identify areas for improvement. By following these strategies, countries can effectively manage medicine waste and protect the environment.

## 4 Conclusion and future needs

Pharmaceuticals have been detected in various parts of Ethiopia, including wastewater, effluents from treatment plants, rivers, lakes, groundwater, and the food chain, including dairy and poultry animal products. The easy availability of many drugs without prescriptions and poor regulation of antibiotic use in the country has likely contributed to the increased presence of pharmaceutical residues in the environment. This issue is further exacerbated by the lack of efficient wastewater treatment facilities, inadequate regulatory frameworks, and insufficient political commitment to enforce environmental protection standards. Factors such as failure to adhere to recommended drug withdrawal periods, low awareness of drug residues, and the availability of prescription antibiotics over the counter among veterinary farmers have been cited as contributing to the environmental presence of pharmaceuticals in Ethiopia. Major water bodies have been contaminated by pharmaceutical effluents from manufacturing industries and healthcare facilities. If these residues remain unregulated, as is the current situation, their levels may increase, posing greater risks to the environment and public health. Given the findings of various studies regarding pharmaceutical residues and the associated regulatory challenges in Ethiopia, it is recommended to focus on addressing antibiotic residues, combating antimicrobial resistance, and mitigating the inappropriate use of antibiotics in animal production.

The government and associated veterinary medicine regulatory bodies must strengthen capacity and establish clear procedures and strategies to monitor and control the irrational use of antibiotics, as well as to manage drug residues in dairy and poultry farm products. There is an urgent need for responsible authorities to eliminate antibiotic misuse and enforce restrictions through an approved list of drugs regulated by government authorities. Safety guidelines must be provided to ensure that edible tissues and other animal products intended for human consumption are treated with drugs that are safe for humans. Regulators should make a strong effort to promote responsible use by emphasizing the importance of adhering to maximum residue limits and withdrawal periods. These measures are critical to ensuring food safety and protecting public health.

The Environmental Protection Authority, in collaboration with the national standard body, should prioritize the development and enforcement of guidelines and standards for the treatment and disposal of pharmaceutical manufacturing effluents. The national medicine regulatory authority (EFDA) must also focus on raising awareness about the proper disposal of household medicines and establishing a drug take-back system to prevent improper disposal practices. Moreover, there should be stringent control over wastewater effluents from healthcare facilities. Healthcare facilities must regularly monitor pharmaceutical residue concentrations and implement effective parameters to minimize their environmental impact. These measures are essential to safeguard the environment, protect water resources, and promote public health.

### 4.1 Recommendations for improvement

This document calls for the establishment of robust waste management systems, enhanced regulatory frameworks, and community education initiatives to ensure compliance with environmental policies. It also highlights the importance of collaboration among stakeholders to address the challenges of pharmaceutical waste management effectively.
